# Transactions of the Odontological Society of Great Britain

**Published:** 1880-07

**Authors:** 


AMERICAN JOURNAL

OF

DENTAL SCIENCE.

Vol. XIV. THIRD SERIES—JULY, 1880. No. 3.

### ARTICLE I.

### *Transactions of Odontological Society of Great Britain.*

Dr. Walker read the following communication: With your permission, Mr. President, I will allude to the fact that in daily practice we are frequently called upon to give an opinion as to the probable duration of a swelling in the maxillary region. The following case may prove to us how hazardous a hasty opinion may prove to be:

A man, aged 35, consulted me, but apparently with great repugnance and indifference, stating even before he had made a foot's entrance into my consulting room, that if his medical man had not refused attendance until he had consulted a surgeon of our specialty and supported this by a threat of discontinuing his treatment altogether, he would not have come to town, as his teeth had been examined many times before and found quite sound.

On enquiry, I ascertained from the patient that twelve months previously he had experienced great pain in the left side of his face and head. After a few days this was followed by throbbing and dull heavy pain in the whole of the superior maxillary region. In a few weeks from the date of the first attack, pain in the left ear and in the region of the temporal bone supervened and continued for weeks, with swelling over the mastoid cells under the sterno-mastoid muscle; resulting in due time in a large abscess, followed by several smaller ones, in tne same region ; debility, and weakness of the general constitution, consistent with the formation of pus. This was succeeded by occasional indistinct visions in the left eye, which increased until he lost the sight.

He had taken several months' rest with total cessation of business and frequent changes to the seaside, but with no beneficial result.

On examination the left eyeball was of different color to the right; there was no apparent swelling in the region of superior or inferior maxilla. There were citricacies over the mastoid cells, but no accumulation of pus.

The hard palate appeared quite healthy; there was slight redness around the margins of the first and second molors; only very slight fullness between the facial muscles and the maxillary bone; no tenderness on pressure. On careful inspection and examination of the teeth, with the assistance of the mouth mirror, all the teeth appeared sound. No discoloration was apparent, even with a strong reflected north light. On digital examination (with the head firmly grasped) all the teeth were quite firm, except the second left upper molar. In this tooth I could feel distinct motion, but with no visible signs of elongation. On introducing a hard substance between the upper and lower teeeth and applying severe pressure by the heavy closure of the jaws, the second molar jaw gave no sign of tenderness. By isolating the second molar with prepared cotton wool placed around the other teeth, and the injection of cold water with a large syringe, a paroxysm of severe pain was the immediate consequence. On extraction of this tooth, one ounce of pus passed into the mouth through the palatine socket. In this case, the palatine fang of the second molar had perforated the floor of the antrum; obscure caries on the posterior proximal surface of the tooth had generated pus, which ac cumulated in the antrum, then passed back through the fallopii to the ear, and under the sterno-mostoid muscle, pointing to the surface. Or, by absorbing a portion of the hard palate, the pus might have passed through the posterior palatine canal into the spheno-maxillary fosse, then through the spheno-maxillary fissure. The patient made a good recovery with perfect restoration of sight.

The President remarked on the interest of the case and the difficulty of the diagnosis, and complimented Dr. Walker on the clearness with which he demonstrated the probable course of the pus on his prepared specimens.

Mr. Sewell said there was one statement in the very interesting communication which Dr. Walker had just read which he was not disposed to accept without farther evidence, viz., that the pus did actually travel to the petrous portion of the temporal bone by way of the canals which Dr. Walker had enumerated. He thought it much more probable that the pus had passed along the bones externally, burrowing in the usual way under the muscles and fascia; especially since there had been no ifntererence with the function of hearing which would probably have happened, had the bone been the seat of an abscess. So, also, he thought that the effects of the eye might be more easily explained by supposing that the pus in the antrum had exerted upward pressure on the floor of the orbit. Had this been the case, considerable disorganization of the contents of that cavity must have resulted, and there would not have been such a rapid recovery after the evacuation of the matter; whilst, on the other hand, very similar symptoms might be observed as the result of pressure in cases of tumor of the antrum. But these criticisms did not in the least diminish the interest in the case, nor could they lessen the credit due to Dr. Walker for having correctly diagnosed such an exceedingly obscure case.

Mr. Gaddes remarked that the influence of the nervous system in exciting purulent secretions on parts remote from one another must not be lost sight of. It might be that in this case the presence of pus in the neighborhood of the ear had been due to the transmission of a reflex nervous impulse, and not to the actual travelling of matter from the superior maxilla to the temporal bone.

Dr. Walker said that a detailed reply to Mr. Sewell's criticisms would take up more time than could be spared that evening, but he might state that this was not the first case of the kind which he had met with. On several occasions patients had been sent to him by ophthalmic surgeons, who had detected the presence of pus in the orbit and had found it to be due to disease of the teeth or palate. He would, on a future occasion, give particulars of four or five cases in which pus had traveled to considerable distances through the bony canals of the skull, and he thought he should be able to convince Mr. Sewell that what he had supposed to have occurred in this case was neither impossible nor improbable.

Mr. Magor showed a left upper wisdom tooth which had been sent by his father for presentation to the Museum. It was very small and deformed, resembling a supernumerary rather than a third molar. The patient, a lady of about forty years of age, had suffered for some time from severe neuralgia of the left side of the head. This tooth was found to be carious; it was extracted and the pain ceased.

Mr. Brown Mason then related the following case: A young man running across a wet lawn slipped and fell with great force, striking the edge of his upper incisors on the edge of a stone step. When he came to Mr. Mason the left upper central was found to have been driven quite up into the alveolus; the right central had a portion of its crown broken off; the outer plate of the alveolus was fractured from canine to canine; the left lateral, although still in its socket, was so loose as to threaten to fall out on the slight est touch, and the other incisors were very loose. Mr. Mason brought down the central incisor with a pair of forceps, and obtained a very fair impression of the whole upper jaw in godova wax, taking care that the teeth should not leave their sockets during the operation. He then made a temporary splint of the same material to keep the teeth in place during the three hours necessary to produce a splint of celluloid. This was made to fit closely to the lingual and labial surfaces of the incisors and canines, and passed back and round the last tooth on each side of the jaw; it was re. tained by ligatures tied to the second biscupid on one side, and to the first (the second being absent) on the other. The accident occurred on February 18th, and the patient had since progressed very satisfactorily; of course, he was still wearing the splint, and would continue to do so until the teeth were quite firm in their sockets.

The President then called upon Dr. Lauder Brunton to read his paper.

### ON NERVOUS DISEASES CONNECTED WITH THE TEETH—BY T. LAUDER BRUNTON, M. D., F. R. S.

*Mr. President and Gentlemen:*—The pain of toothache localized in a decayed tooth is unfortunately so common that every sufferer diagnoses it for himself, and although it may be reckoned among the nervous disorders connected with the teeth, I need not say anything about it.

But toothache may be associated with other pains, or may even be replaced by them, and then the diagnosis is by no means easy. The true cause of the pain may, indeed, remain unsuspected even by competent medical men, and their treatment may consequently be comparatively ineffectual. My attention was first drawn to the connection between decayed teeth and nervous disorders having little or no apparent relation te them dy an incident which occurred a good many years ago, when I was a student. I had just heard that one of the best means of relieving toothache was to insert a pledget of cotton wool, dipped in melted carbolic acid, into the cavity of the aching tooth, care being of course taken to squeeze out the superfluous acid and to cover the pledget with some clean wool so as to protect the tongue. I was very anxious to test the information I had received, and shortly afterwards an opportunity presented itself. A

maid servant had complained for several days of headache in the left temple of a severe neuralgic character, and associated with this was a certain amount of toothache, which was, however, less complained of than the headache. I plugged the offending tooth with cotton wool dipped in melted carbolic acid, but was greatly disappointed to find that it produced little or no apparent benefit. In less than half an hour, however, the girl informed me that the pain in the temple and the toothache were both entirely gone. Their disappearance was not due to the carbolic acid having acquired time to exert its action, but to its having been applied to a different point. The girl had taken it ont of the cavity of the decayed molar into which I put it at first, and transferred it to another tooth, of which she had not complained, and which I had not suspected. Immediately the pain disappeared, both from the tooth and temple.

In this case the pain was felt in the tooth as well as the head, and the headache might be looked upon as simply irradiation of the pain from the tooth. But the headaches may occasionally depend upon caries of the teeth in which no pain whatever is felt, is, I think shown by what once happened in my own case. I had been suffering from migraine, the pain being limited to a spot in the left temple. There was tenderness on pressure on one spot below, and in front of, the parietal eminence. On several occasions I had noticed that the left eyeball was tender on pressure; but one day I was suffering from headache, and yet found that the eyeball was not tender. I pressed my finger all over my face in the endeavor to find a second tender spot, and at last I found one under the angle of the jaw. But the tenderness here was due to a small gland, which was hard and painful to the touch. Hardness, enlargement and tenderness in a gland generally indicate more or less inflamation in it, and the most probable cause of such a condition is, of course, the irritation excited in the gland by foreign matter conveyed to it by the lymphatic vessels. I accordingly began to examine the mouth and teeth from which the lymphatic vessels proceeded to the gland in question. Nothing abnormal was to be noticed in the lips, cheeks, tongue or gums, so I tested the teeth by percussion with a blunt steel point, and on the posterior aspect of the last molar on the left side of the lower jaw I found a spot which was very slightly tender. I accordingly went to a dentist, and learned that caries had just begun at that spot, but had not caused any cavity whatever. I had never suffered the least pain in the tooth, and but for the headache which led me to percuss the teeth systematically, I should, in all probability, never have suspected the caries until it was far gone. The connection which was here found to exist between temporal headache and a decayed tooth is, I think, interesting, not only as showing a casual relation between the caries and the headache, but as helping to explain the phatology of migraine.

A good deal has been written on this subject, and there is a considerable diversity of opinion amongst different writers. Professor Du Bois Reymond, who suffered a good deal from it, attributed it to spasm of the vessels for he found that, during the pain, the temporal artery became terse and hard, like a piece of whip cord, and the pupil of the eye on the affected side dilated as if the sympathetic in the neck had been irritated Others have discarded this explanation, because they found that the vessels, instead of being firmly contracted, were distended widely and throbbed violently, and they have attributed the pain in the head to the congestion of the vessels.

These two explanations of the pain of migrain, the one attributing it to anæmia, and the other to congestion, are apparently irreconcilable. My own case gives, however, I think, an explanation of the discrepancy. Both statements are currect, but both are incomplete, and the reason is that their authors have only observed the arteries during a part of their course, instead of tracing them backwards to the large trunks from which they sprang and onwards to their smaller ramifications. In my own case, I have found that on some occasions the temporal artery was hard and contracted like a piece of whip cord, as described by Du Bois Reymond. On others I found the temporal artery widely dilated and pulsating violently, and yet I could distinguish no dsfference between the pain I felt on these different occasions. So not contented with noting the condition of the temporal artery only at its middle, I followed it onward to its smaller branches and backwards to the cartoid.

Then I found that a constant vascular condition existed during the headache, notwithstanding the apparent differences in the state of the temporal artery. This constant vascular condition consisted in dilation of the artery at its proximal, and spasmodic contraction at its distal extremity. The carotoid artery was almost invariably dilated and throbbing. Sometimes the dilation would extend as far as the trunk of the temporal artery, but sometimes the temporal was contracted. Even when the temporal artery was dilated, if one only followed it to its smaller ramifications, they were found to be firmly contracted and cord-like. If one may reason from this singular instance, connecting as it does the examples of vascular dilation and eontraction given by other authors, we may say that the pain of migraine depends neither on contraction nor dilation of the vessels *per se*, but upon dilation of the one part of the vessel with spasmodic contraction of another, or, if we might so term it, upon a state of colic in the vessels themselves. This irregular contraction of the vessel is almost certainly due to disordered vaso-motor innervation. The cause of this disorder is to be sought in the sympathetic systsem, and the observation of Du Bois Reymond regarding the condition of the iris may lead us to connect it with the cervical ganglia. From these ganglia vaso-motor fibres proceed along the carotid and its branches, and if we regard disorder of these ganglia as the cause of migraine, we are at once in a posiition to explain some of the symptoms which occasionally accompany it. Thus, I have observed that sometimes the pain in the temple would suddenly cease and be replaced by pain in the occipital region. Sometimes, also, we have affections of the sight, such as general dimness of vision, diplopia and spectra—colored or uncolored. The transference of pain from the temple to the occipital regoin is probably caused by transference of the spasmodic contraction from the temporal to the occipital artery, and the disorders of the sense of sight we may reasonably regard as caused by alterations in the intercranial branches of the carotid, similar to those which we can detect by the finger in its temporal branch. The disturbance in the sympathetic system which I regard as the curse of migraine, may not always have its origin in the teeth; it may, and very probably does, sometimes originate in the eyes, but in the instance I have already noted as occurring in my own case, the irritation started from the lymphatic gland, on or about which branches es of the sympathetic probably ramified. The tooth itself, although the real cause of the sympathetic irritation, did not produce it directly, but indirectly. From the root of the tooth the lymphatics conveyed irritating matter to the gland, and the irritation here excited acted in its turn as a disturber of the sympathetic nerves which furnish the vasomotor supply to the carotid and its branches.

The connection between dental caries and neuralgia was first noticed by Neucourt, and he gives rules for diagnosing a casual relation between caries and neuralgia. When the pain, which is at first widespread, gets localized in the course of a few days in the dental region, and is succeeded by redness, swelling tenderness on pressure of the gums, the neuralgia is almost certainly of dental origin. In these cases the patients are restless, and the pain is more or less constant, with no distinct intermissions; the pulse is more frequent and hard, and there is not unfrequently sweating. If the pain is followed by a gumboil, the tooth, he thinks, is certainly decayed, although it should present no appearance of caries, and this he considers to be also the case if the tooth appears to be longer than the others and is painful on percussion. Tenderness on percussion is considered by Richter to be the most certain sign. The diagnosis may be assisted by noticing whether the neuralgia when disappearing lingers longest in one of the teeth.

The exact pathology of neuralgia has not yet been settled, but Valliex, one of the great authorities on the subject, gave as its distinctive points the presence of spots which were tender on pressure and the effect of pressure in increasing pain. These spots have been noticed by Neucourt in neuralgia depending upon dental irritation, and he has also observed the absence of increased pain on pressure in true neuralgia, so that no distinction can be drawn between neuralgia due to dental irritation and neuralgia depending upon other causes.

Although the most frequent seat of pain due to carious teeth is the temporal region, yet as one would expect, we find it also in parts of the neck. A few weeks ago I was consulted by a lady regarding her throat. She had pain opposite the upper part of the thyroid cartilage on the right side and thought that she had inflamation at that point. Laryngoscopic examination showed the larynx to be perfectly healthy, but I found one of the molars on the same side as the painful spot to be extensively diseased. The pain from which she suffered, I have little doubt, was caused by the decayed tooth, but, as she refused to have it extracted or stopped, I could not absolutely verify my diagnosis. I put her upon a course of tonics and the pain almost completely disappeared.

This would be said by some to prove my diagnosis to be wrong; for if the pain depended on the presence of a carious tooth, how could it disappear while the tooth remained unattended to? But we must always remember that the actions which take place in the animal body are not so simple as those which occur in the test tube of a chemist. Yet, even in the test tube we require more than one reagent to produce a reaction; and if one of the substances or conditions necessary for the reaction be absent, it does not occur, even though other conditions be present. In the same way we know that a decayed tooth does not always cause toothache, and that toothache, when present, may frequently be removed by the use of a saline purgative. the tooth still remains as a source of irritation, but the state of the nervous system has been so altered by the purgative that pain is no longer produced by the irritation. In the same way we may not unfrequently relieve the neuralgia, originating from decayed teeth, by a judicious course of aperients and tonics. This is so far advantageous to the patient, as it relieves him from pain: but it is, on the other hand, disadvantageous, in as much as it causes the medical man to overlook the real source of evil, and allows the dental caries to proceed, instead of having it arrested by suitable stopping. In the case I have just mentioned, the pain in the larynx, which I attributed to the decayed tooth, did not lead to any change in the nutrition or functions of the larynx. Pointis, however, records a case in which after severe toothache, the patient suddenly lost his voice, and the aphonia was followed by anorexia, cough, wasting and feverishness, which led to the belief that he was suffering from laryngeal phthisis. But the lungs were sound and there was no tenderness over the larynx. There was slight inflamation of the pharynx and all the molars on the left under jaw were decayed, and the gums and periosteum around them were swelled. The teeth were removed, the gums cauterized, and the gargles employed. On the very day the teeth were extracted, the suffocative spasms which had troubled the patient abated; on the following days the other symptoms quickly disappeared.

The irritation caused to the larynx by the process of dentition is well recognized, and has led to the employment of the term teething-cough. The existence of a real casual connection between cough and teething has been doubted, but there are cases on record that seem to show that this really does exist. One very marked instance of this sort has been recorded by Paasch. A child, four months old, had a paroxysmal laryngeal cough, during which it was nearly suffocated, opening its mouth and throwing the head back. Narcotics were of no use. The gum of the lower jaw was swelled, and vesicular swellings appeared at the part of the gums corresponding to the middle incisors. These increased in size, and became dark, livid, translucent and fluctuating. During their growth the cough increased, but when the right incisor came through the gum, and one vesicular swelling broke, the cough ceased. After twenty-four hours it again began, though less violent than before. After some days the second incisor came through, the second vesicle burst, the cough at once began to disappear, and at the end of two days had entirely and forever gone.

From the close connection that exists between the throat and the ear we would expect deafness to be not unfrequently the consequence of dental irritation. It seems, however not to be very frequent, although it does exist, as shown by the following case, recorded by Koecker. A man, aged forty-eight, suffered from suddenly increasing deafness, but after his teeth, which were carious, and caused supportion, were extracted, he completely regained his hearing.

The eye is much more frequently affected than the ear, and blindness is by no means an uncommon result of dental decay. Mr. Jonathon Hutchinson has recorded some cases of this, and he regards the blindness as reflex, and analogus in its causation to essential paralysis of children. The sight is suddenly lost but there are no cerebral symptoms. The optic nerve is sometimes athropied, but sometimes not. The blindness is generally preceded for a long time by facial neuralgia, associated with toothache. A more striking case than any of Mr. Hutchinson's is recorded by Dr. De Witt. A perfectly healthy man, aged thirty-one, suddenly noticed, in attempting to fire off a gun, that his right eye was completely blind. He had neither pain nor subjective appearances of light in the eye. He was able to distinguish light from darkness with it, but nothing more. No cause for this blindness could be discovered until twelve years afterwards, when it was found that the patient had several teeth stopped two months before his blindness. For a long time afterwads he suffered from pain and tenderness in the first molar of the right side. The gums swelled and ulcerated, and frequent abscesses formed, which he opened with his knife. The stopping was at length removed from the tooth, and this at once relieved the irritation of the gums and increased the power of sight. In three weeks, however, when the sight had become considerably better, the gums again ulcerated and the sight became immediately worse. The decayed tooth was then extracted and the sight became permanently improved, although it never became quite so good as that of the other eye.

The connection between the teeth and the sight has long been popularly recognized in the name of "eye teeth" given to the cannies, and this seems to depend on no popular superstition, but on a real scientific fact. It is believed by many that the extraction or decay of a canine leads to loss of sight, or inflamation in the corresponding eye, and the physiological experiments of Magendie and Schiff substantiate this belief.

Magendie divided the inferior maxillary branch of the fifth, and Schiff divided the lingual and inferior dental branches, without injury to the ophthalmic branches. The dimness of vision produced by these experiments is referred by Schiff to disturbance of the vaso motor supply to the eye consequent upon a partial analysis of the ophthalmic branch of the fifth; but as this nerve itself is not injured in the experiment, it is evident that the vascular alterations are of reflex origin, the irritation having been conveyed from the sight of the wound to the nerve centres, and having there exerted such an influence upon them as to induce vascular changes in the eye.

The eyelid may also be affected reflexly from the teeth. Sometimes dental irritation may cause motor spasms, and at other times paralysis. A year or two ago I had the stump of a bicuspid tooth extracted from the right upper jaw. Almost immediately after the extraction I noticed a con stant spasmodic twitching in the right eyelid, which I was utterly unable to restrain. This lasted all the time the wound in the gum caused by the extraction of the stump was open, but it ceased as soon as the gum had healed and has never since returned. A case is recorded by Gaine in which a carious tooth in the upper jaw had caused an abscess in the antrum. The right upper lid was paralyzed, the pupil dilated, and there was no reaction. The optic nerve was pale and the eye blind. On extraction of the tooth and puncture of the antrum the paralysis of the lid disappeared, although the eye did not regain sight.

Spasmodic contraction of the masseters is another consequence of dental irritation. A few weeks ago a gentleman, over forty years of age, called upon me, and told me that he was much concerned about a spasmodic affection of the jaw from which he was suffering. He was, in fact, afraid of lockjaw. He felt obliged to keep his mouth open, because it seemed to him that if he once shut it he would not be able to open it again. I did not recollect having read any description, but it seemed evident that it must depend either upon congestion of the cerebral centre for the movement of the jaw, which Ferrier locates at the lower end of the fissure of Rolando, or on reflex irritation from the mouth itself. The latter seemed to be much the more probable, and on looking into his mouth I saw that the teeth did not seem to be altogether in good order. I accordingly requested him to see a dentist, and on inspection the source of irritation was discovered to be a wisdom tooth, which was just making its way through the gum, but in a somewhat oblique direction, so that its crown was pressed against that of the molar in front of it. On looking up the literature of the subject, I discovered that this affection was pretty fully described by Germain, who recognized two causes for this orm of trismus. The first is when the back molar is decayed and a gum-boil forms at its base, and the other is when the attachment of the masseter extends in front of the angle of lower jaw and wisdom tooth, in appearing must break through its muscular and fibrous attachment. Colin states that every year he sees at least one perfectly healthy individual become suddenly affected with spasmodic contraction of the masseters. There is no fever, but the contraction is so strong that only fluid nourishment can be taken. The contraction can be felt by running the finger over the masseter muscle. It gradually disappears in about eight or fourteen days. Little treatment is required, except attention to the bowels, and possibly, if the contraction be very severe, an injection of atropia into the muscular substance itself might be of service.

We have already noticed paralysis of the eyelid as a consequence of dental irritation, and we have also discussed the pathology of temporal and occipital headache in relation to caries of the teeth. Sometimes, however, paralysis occurs of a much more extensive character, in consequence of dental irritation, especially in children. Teething is recognized by Romberg and Henoch as a frequent case of analysis appearing in children without any apparent cause. According to Fliess, paralysis of this sort occurs more commonly during the period of second dentition, whereas convulsions generall occur during the first. Its onset is sudden. The child is apparently in good health, but at night it sleeps restlessly and is a little feverish. Next morning the arm, or more rarely the leg, is paralyzed. The arm drops; it is warm but swollen, and of a reddish blue color. It is quite immovable, but the child suffers little or no pain. Not unfrequently paralysis is preceded by choleric movements. Sometimes recovery is rapid, but at other times the limb atrophies, and the paralysis may become asssociated with symptoms indicating more extensive disturbance of the spinal cord and brain, such as difficulty of breathing, asthma, palpitation, distortion of the face and squint, ending in coma and death.

It is only in very rare instances that we are able to gain any insight into the pathological anatomy of such cases, because they rarely prove fatal, and even when they do so, the secondary changes are generally so considerable as to leave one in doubt as to the exact mode of commencement. This renders all the more valuable the case recorded by Fliess, in which a boy five years old, and apparently quite healthy, found his left arm completely paralyzed on waking one morning after a restless night. The arm was red, but the boy suffered no pain, and played about without paying much attention to the arm. The same day he fell from a wagon upon his head and died in a few hours. Apart from the fracture on the skull, which caused his death, the anatomical appearances which were found, were congestion of spinal cord near the point of origin of the bronchial nerves; the meninges were here much reddened and congested; the veins were much fuller than the corresponding right side. There was no organic change perceptible, either in the spinal chord or the bronchial nerves. On the other hand, the turgesence of the veins extended from the shoulder and neck up to the face, and was very striking in the submaxillary regino.

This vascular congestion seems to point to vaso-motor disturbance of a similar kind to that which we have already noticed in connection with occipital headache, or with migraine accompanied by subjective appearances of either form or color. Chronic movements, as we have said, have been noticed as prodromata of paralysis, and occasionally dental irritation may give rise to cholera alone. This irritation may depend, according to Levick, either upon the sound dentition, or upon dental caries, and the casual connection between the two is shown by the fact of it disappearing when the tooth pierces the gum, or when the carious teeth are extracted.

According to Russell Reynolds, the second dentition is also a cause of epilepsy, and he has observed that those who are affected by it have often suffered from convulsions during the first dentition. A case is recorded by Albrecht of a boy, aged twelve, who suffered daily for twelve months from general convulsions, which began in the temporal region and extended to the external auditory meatus. There was no decay in this instance, but the teeth were large, and the last molar on the right side had its crown jammed into the ascending ramus of the jaw. As soon as it was extracted the pain ceased, and the convulsions did not return. Another case is given by Mr. Castle of a young man, aged nineteen, who had complained for four years of headache and pain in the eyes, stiff neck, swelling and numbness of the right arm. For the latter two years he suffered from general convulsions, which came on every two or three days ending in vomiting, and often succeded by partial deafness. All treatment was useless, and setons and blisters to the neck did no good. Nearly all the teeth were decayed; nine of them were extracted, and almost all of them had matter at their roots. A gargle was given with five grains of iodide of mercury twice a day, and a purgative twice a week. After the extraction of the teeth the fits entirely disappeared.

Affections of the intestinal track depending on dental irritation are of very considerable importance indeed. The diarrhœa which comes on in children during dentition is well known, and is probably of a reflex character.

In adults many a case is due to defective teeth, partly, it may be, from a reflex affection of the nerves, both secretory and motor, of the stomach and intestines, but partly also, without doubt, from the imperfect mastication of the food, which is swallowed without being broken up on account of the pain or inconvenience which the act of mastication causes. In this way two evils are occasioned. First of all, the shortened sojourn of the food in the mouth allows no time for the secretion of saliva. From want of this the starchy constituents of the food are imperfectly digested; and, moreover, deficiency of saliva also lessens the normal stimulus to the secretion of the gastric juice; for alkaline fluids, like saliva, stimulate the secretion from the stomach, and this deficiency of saliva is accordingly followed by a deficiency of the gastric juice. But, secondly, imperfect mastication has a mechanical action in preventing perfect digestion, for the food, being swallowed in lumps, is not permeated by the digestive fluids, and thus cannot be dissolved in anything like the same period of time that it would otherwise be. The diarrhoea which occurs in children is probably produced through the gastric and intestinal branches of the vagus, and other branches of this nerve may be affected reflexly from the teeth. In a case recorded by Lederer, the second left upper incisor was replaced in a young girl by an artificial tooth. Shortly afterwards she became ill, vomited everything, and suffered from convulsions. No remedy succeeded until the tooth was removed and shortened. Immediately all the symptoms from which she had suffered disappeared.

The close connection between the roots of the fifth nerve, and those of the vagus, can be demonstrated anatomically, and it is probably in consequence of this that irritation of the fifth is able to exert such a powerful influence upon the circulation. Some time ago, in a paper which I published in the *British Medical Journal*, I mentioned that the cause of death during the extraction of teetn under chloroform, was probably the stopp ge of the heart's action through the inhibitory fibres of the vagus, associated with a reflex depression of tone in the blood vessels. The reason why the extraction of a tooth in a person who is not under the influence of anæsthetic is followed by no ill effects, is probably this: that in him the irritation of the fifth nerve produces two distinct actions which counterbalance each other. It may cause reflex stoppage of the heart through the vagus, but at the same time it causes reflex contraction of the vessels through the vaso-motor centre. This contraction of the vessels maintains the pressure in the arterial system during the stoppage of the heart and thus no harm whatever is done. When anæsthetic is used, however, one of these pieces of nervous mechanism may be paralyzed by it, while the other is not, and the extraction of the tooth may stop the heart without causing contraction of the vessels. The blood-pressure will then sink very rapidly in the arterial system, and fatal syncope may be produced. If, however, the anæsthetic be pushed to a greater extent, so that both parts of the nervous mechanism just mentioned are paralyzed, the vessels are not contracted; but neither is the heart stopped. The operation, is therefore, comparatively free from danger when no anæsthetic has been given, or when the anæsthesia is perfectly complete, the period of danger being that of imperfect anæsthesia.

We have seen how affections of sensation, of motion, and of nutrition may all be dependent upon dental irritation, but even the cerebral faculties themselves may also suffer from a similar cause. One or two very interesting cases of this sort are recorded by Dr. Savage in the *Practitioner* for June, 1876. The first of these was that of a farmer, aged twenty-two, with a strong family tendency to insanity. In May, 1875, he suddenly took to riding madly about the country without his coat or waistcoat. From May until November he was exceedingly noisy, destructive, untidy, almost constantly excited, and if for a day or two he was exhausted, he was sullen and more dangerous. In the middle of November he complained of very severe toothache that caused him to be sleepless. He bore this for two or three days, after which the stump was removed. There was suppuration at the root of the fang. From the time that the stump was extracted the patient steadily improved, and by the middle of December was quite well. Another case was that of a woman, aged thirty-four, who had a brother insane and had herself been intemperate. She was admitted in September, 1875, suffering from acute mania. She was noisy, violent and obscene. She continued to be so until January 20th, 1876, when she complained of great pain, with swelling and redness of her right lower maxilla. She had some bad teeth, but did not complain of toothache. The pain and swelling increased, and at the same time she became quiet and reasonable. She said she could not remember much of her state of excitement. The swelling of her face subsided and she remained quite well. This case, however, was not so convincing as the first one recorded, because here there was a second possible cause of recovery, as she was pregnant, and said she felt quickening about ten days before her recovery. The recovery, however, was coincident with the pain and swelling of the face, and seemed, rather than the quickening, to be the cause of the recovery.

The President said he thought members of their profession might congratulate themselves on the fact that medical men now paid more attention than formerly to the effects of local irritation. He had met with many cases in which the patient might have been saved much suffering had some of the facts to which Dr. Brunton had called attention been more generally known. He remembered especially the case of a gentleman whose health had been greatly impaired by severe neuralgia so that when he came to Mr. Woodhouse he was reduced to living on brandy and milk, and was miserably thin and weak. Mr. Woodhouse found that both upper canines were carious, the pulp of one being exposed and inflamed, though the patient never had any pain in it; the other was not so bad. He destroyed the pulp of the one and filled the other. The patient had no pain afterwards and soon regained his health. He hoped that Dr. Brunton's paper would again draw the attention of medical men to this important subject.

Mr. Charles Tomes said he wished in the first place to ask Dr. Brunton a question. He had referred in his paper to some experiments which had been performed by Prof. Schiff, in which he found that irritation of the branch of the fifth nerve supplying the teeth produced a reflex effect on the eye of the same side. Mr. Tomes would be glad if Dr. Brunton could inform him on what animals these experiments were tried and in what way the impairment of vision was tested. The correctness of these observations was of some practical importance, since division of the inferior dental nerve was a recognized surgical operation which was practiced as a last resource in cases of obstinate neuralgia. He had performed the operation a good many times and had never known it to be followed by dimness of vision, but if it should appear that there was some risk of this occurring, of course the operation must be abandoned.

Then, with regard to the general pathology of migraine. Dr. Brunton had advocated the view that it was due to spasmodic contraction of the vessels. If this were so, the pain should always be relieved by nitrate of amyl, but this was not invariably the case, even though the nitrate was so administered as to produce extreme vascular relaxation. The inhalation of ether again produced great vascular relaxation, evidenced by flushed face, full bounding pulse, etc., but on one occasion he had distinct evidence of the occurrence of an attack of neuralgia whilst the patient was fully under the influence of ether. This was in the case of a man who suffered from very severe paroxysmal attacks of neuralgia affecting the region of the inferior dental nerve and always accompanied by a spasmodic twitching of the lip. Dr. Tomes first tried stretching the nerve at the mental foramen, and as this gave no relief, he cut down upon it again and removed a portion. During one of these operations, whilst the patient was fully etherized, the peculiar twitching of the lip occurred which indicated a paroxyism of neuralgia.

Lastly, Dr. Brunton had quoted some German authority who asserted that the spasm of the masseter which was not unfrequently set up by the wisdom-teeth during the process of eruption, was caused by the tooth tearing through the fibrous attachment of the muscle, and that it was thus due to direct, and not to reflex, irritation. But the tooth, if erupted in the usual situation, did not approach anywhere near the masseter: and if the tooth was displaced outwards, the external plate of the jaw was so exceedingly thick and hard, that the tooth on coming into contact with it, was always diverted from its former course. He thought, therefore, that the view usually held, viz., that the spasm was of reflex origin, was more likely to be correct.

*ical Press and Circular.* It should be remembered that Mr. Sewill thought that the Society was to be congratulated on the fact that it would have in its "Transactions" the best account of the connection of the teeth with nervous disorders which had yet been published. Dr. Brunton had collected from various sources some remarkable cases of serious disorders of the nervous system resulting from dental irritation, but he himself had been greatly impressed by the extreme rarity of such cases in practice. He had been for twelve years dental surgeon to a metropolitan hospital; he had seen during that time not only a large number of patients who had applied to him directly, but had also had a considerable number of patients referred to him by his medical and surgical colleagues, and he had kept careful records of all exceptional cases. Yet among all these cases he had not met with a single case of nervous disorder which could be said to be directly dependent on diseases of the teeth. Some nervous diseases such as epilepsy, might be aggravated by dental irritation, but as to the direct result of this he had met with nothing more serious than neuralgia and spasm of the masseter. He had not even met with a case of distant neuralgia which could be assigned to this cause; the pain was, in his case, always confined to the head and face. He might remark, also, that Dr. Brunton's personal experience was exceptional; *incipient* caries rarely produced neuralgia; its occurrence generally indicated the existence of a large cavity. Nor did he think that incipient caries would cause enlargement of a lymphatic gland. If this was present, inflammation of the pulp, alveolar abscess or periostitis would almost certainly be found. On the other hand, there was no doubt that dental neuralgia might intermit, although the exciting cause was still present, and also that it could be cured, temporarily at least, by tonics. An interesting case of spasm of the masseter, caused by dental irritation, had lately been related by the President of the College of Surgeons, of Ireland, at a meeting of the Surgical Society of Ireland, and would be found reported in the *Med-*

a plate by an epileptic was dangerous, he did not remove inflammation of the parts about the jaw might cause a stiff ness which would closely resemble spasm of the masseter, and, as a matter of fact, inability to open the jaw was more often due to inflammatory changes than to nervous spasm. The remarks he had made respecting the rarity of severe nervous symptoms as the result of dental irritation referred only to adults. In infancy grave nervous disorders did no doubt occur not unfrequently from this cause, but dentists did not as a rule see much of this class of cases.

Mr. Coleman said he was surprised to hear that Mr. Sewill had never met with cases of nervous derangement which were due to dental irritation. He himself had published several very clear cases in the *Lancet* some years ago and had seen many since. Quite recently a little girl was brought to him who had become subject to fits; he removed some carious teeth and the patient had no more fits. The late Mr. Holmes Coote had assured him that talipes equinius was more frequently due to nerve irritation set up during teething than to any other cause. He would take the opportunity of thanking Dr. Brunton for his paper, which, if it did not set forth any new facts, certainly gave a more scientific explanation of the pathology of these cases than had yet been attempted.

Mr. Gaddes said he had lately met with a case which showed clearly the effect of dental irritation in aggravating nervous disease. A girl, aged seventeen, came to him at the National Dental Hospital; she had been subject to epiliptic fits for two years, and when she came to him she was having on an average two a week. There was no family history of epilepsy. Some of the bicuspids were much broken down by caries and had decomposing pulps. Some time previously she had broken her upper central incisors in one of the fits, and the pulps of these teeth also were exposed and suppurating. As the girl had some pretensions to good looks, which would have been materially affected by the extraction of the teeth, and the wearing of them, but cleared out and filled the pulp canals. The result of this treatment was that during the next three months the girl had but one fit, showing an evident connection between the state of the mouth and the fits.

The President here called attention to the fact that the hour at which the meeting usually terminated had almost arrived. He thought, however, that considering the interest and importance attached to the subject under discussion, the members present would probably wish to prolong the debate for another half hour.

A motion to this effect having been proposed and seconded by Mr. Oakley Coles and Dr. Walker, was put to the meeting and carried unanimously.

Mr. Oakley Coles said he thought every dentist must have met with cases like those referred to by Mr. Coleman and Mr. Gaddes. Not long since a girl was brought to him who had become subject to epileptic fits; he extracted two very carious teeth and had no fit since. He was much surprised to hear Dr. Brunton's report of the result of Prof. Schiff's experiments on the inferior dental nerve. He himself had never known any impairment of vision follow its division. He remembered a case in which the late Sir William Fergusson divided this nerve fourteen times on the same patient; after each operation the man obtained relief from pain for some weeks, or even a few months, and he certainly never made any complaint of dimness of sight.

Dr. Walker said he had seen many instances of the serious effects of dental irritation on the nervous system of young children, especially when they were weakly or had a scorbutic tendency. He would especially instance one family, all the surviving members of which had been for some years past constantly under his observation. The first three children died during the period of first dentition; after this, orders were given that the others should be taken to the dentist as soon as they were six months old, and afterwards whenever they suffered any little indisposition. Since these regulations had been carried out seven children had been successfully reared. The children were brought to him directly any symptoms of irritation showed themselves; he never lanced the gums, but scarified them freely with a sharp point. On several occasions the good effects of this treatment had been most marked. For example, the father going home to his country-seat, on Saturday, noticed that one of the children squinted; he was sent to Dr. Walker on Monday, the squint still persisting, but next day it had entirely disappeared. Another child lost the use of one leg for three days; the gums were scarified and he was well again directly.

About nine years ago a child was brought to him who had been for some time subject to epileptic fits. Her general health was very bad and her teeth much decayed. She remained under treatment fifteen months, and as the mouth was got into better order the fits gradually disappeared, and she had since turned out a very clever girl.

A member said he had met with a very similar case. A boy, aged nine, was brought to him by his mother, who stated that he had suffered for ten months past from frequent fits. On examining the patient's mouth, he found that the second dentition was backward; a lower molar was also decayed, though not much, still the boy said he had suffered occasional pain in it. He filled this tooth and removed the deciduous left lower lateral and canine. A fortnight afterwards the patient returned much improved in health and from that time had no more fits.

Mr. F. Canton said he should like to ask Dr. Brunton whether he thought that paralysis of the arm or leg could occur in an adult as the result of dental irraitation? He had met with a case in which pain in a lower molar had been accompanied by spasm of the muscles of the arm and leg, followed by partial paralysis. As the patient was an anæmic young lady, and the effects seemed disproportionate to the cause, he looked upon the affection as being due to hysteria. At the same time he took care to impress upon the patient the idea that the tooth was the cause, and that its extraction would be followed by a complete dis-appearance of the paralysis. This did actually come to pass, but he did not feel at all sure as to the mode in which the cure was affected.

Mr. J. S. Turner said his own experience enabled him to answer Mr. Canton's question in the affirmative. About two years ago a gentleman, aged twenty-eight, was brought to him from the country. The patient came into his consulting room held up on either side by his father and brother, being unable to walk, and sometimes even to stand, without this assistance—he had also severe trismus. The cause of all this was a lower wisdom-tooth which had grown horizontally outward, perforating the strong outer plate of the maxilla, so that the crown was imbedded in the substance of the masseter. The patient was a stout, healthy man of somewhat nervous temperament, but certainly not inclined to hysteria. The tooth was removed with considerable difficulty, and the patient made a speedy and complete recovery.

The following case was also interesting from the difficulty of the diagnosis, the usual order of things being reversed. A lady consulted him on account of periodical attacks of most intense left hemicrania; any great excitement would bring on an attack—as, for instance, when her husband came home from sea—and whilst the pain lasted she was obliged to shut herself up in a dark room, and was unable to see anybody or do anything. She had a most splendid set of teeth and had never suffered the least pain in any of them, but the wisdom teeth were only partially erupted, and as on careful enquiry the pain appeared to be ultimately referred to the upper dental region, Mr. Turner suspected that the left upper wisdom tooth must be the source of irritation. He therefore removed it, and found that it was carious on the buccal surface, the disease having been hidden by the gum. From that day the patient had no more neuralgia.

Mr. Dennant said that had it been necessary he could have confirmed from his own experience nearly all that Dr. Brunton had said respecting the effects of dental irritation on the nervous system, but as so much had been said on this subject he would relate a case that would warn members not to confine their search for decayed teeth as the cause of neuralgia of the head and face, since irritation of other cranial nerves might by reflex action produce very similar results. A lady, of middle age, consulted him on account of severe neuralgia of the head and face, which she thought might be due to a diseased tooth; he examined and percussed the teeth, but could find nothing. A paroxysm of pain came on during the interview, and the patient at once asked if there were any flowers in the room, adding that the smell of flowers always had this effect. There were some roses on the table at the time. Any irritation of the olfactory nerve would bring on the pain—even the smell of smoke. Mr. Dennant recommended mountain air, and she then said that she had suffered in a somewhat similar manner fifteen years before, and that the same advice was then given to her, and that she had derived great benefit from the change.

Mr. S. J. Hutchinson said he should like to ask Dr. Brunton three questions:—(1) Had he any experience of tincture of Hamamelis in the local treatment of facial neuralgia? (2) He had understood him to say that the inhalation of chloroform in small quantity was apt to exert a depressing effect on the action of the heart, but that when given in larger amounts the effect was counterbalanced by contraction of the small arteries: how did the action of nitrous oxide differ from chloroform in these respects? (3) He had referred to the fact that purgatives would cure neuralgia: could he give any explanation of the way in which this was effected.

The President having called upon the author of the paper for his reply,—

Dr. Brunton said that as the time at his disposal was very short he could only deal briefly with the most important of the questions which had been put to him—those especially requiring an answer.

He could not then reply to Mr. Tome's first question, for he had been unable to obtain a copy of the original paper by Prof. Schiff, but he hoped to be able to get one in a few days, and would then give Mr. Tomes the information he required. Mr. Tomes doubted his explanation of the pain of migraine, because nitrite of amyl did not relieve it, although it produced relaxation of the vessels. But the fact was that the nitre did not uniformly dilate; it could not dilate a vessel which was contracted by the irritation of a vaso-motor nerve. Hence, although it produced great *general* vascular relaxation, it might not be able to suspend the reflex action of the vaso–motor nerves, and the *local* contraction due to this cause would persist. The same thing probably occurred during the administration of ether, but this he could not positively assert.

He would admit the justice of some of Mr. Sewill's criticisms, and would make some verbal alterations in his paper before it was printed.

In answer to Mr. Canton, he would say that dental irritation might certainly cause paralysis in an adult as well as in a child. The brain centre which presided over the movements of the hand, and that which governed the mouth, were so closely connected, that excessive stimulation of one centre might easily derange the functions of the other.

With regard to Mr. Hutchinson's question. He had never used the tincture of Hamamelis Virginica for dental purposes; it had been recommended to him as a remedy for the irritation of mosquito bites, and he had tried it, but with very unsatisfactory results. As to the action of chloroform on the heart,—The nerve from the tooth would act upon the vagus centre and upon the vaso–motor centre in the brain. The vagus centre, being irritated, would tend to weaken, or even to stop, the heart's action, but the vaso–motor centre, being equally contracted would cause contraction of the arterioles and thus no fall of blood pressure would occur. But when chloroform was administered its first effect (*i. e.* when given in small quantity) was to paralyze the vaso-motor centre; the action of the vagus being thus left unopposed, cessation of the heart's centre might occur without any simultaneous contraction of the arterioles, there would then be an immediate rapid fall of blood-pressure and fatal syncope might result. Nitrous oxide was opposed to chloroform in that it, or the venous condition of the blood which it induced, acted as a strong stimulus to the vaso-motor centre, and thus the danger from syncope was reduced to minimum. Mr. Hutchinson's third question was one which it would be impossible to answer briefly, since it opened up a very complex and difficult subject, and he would not therefore attempt it.

On the motion of the President, a hearty vote of thanks was given to Dr. Brunton and to the contributors of the casual communications.

The meeting then terminated.

